# Cross-sectional study: long term follow-up care for pediatric cancer survivors in a developing country, Turkey: current status, challenges, and future perspectives

**DOI:** 10.3906/sag-1911-193

**Published:** 2020-12-17

**Authors:** Sonay İNCESOY ÖZDEMİR, Nurdan TAÇYILDIZ, Ali VARAN, Rejin KEBUDİ, Bülent ZÜLFİKAR, Tiraje CELKAN, Gürses ŞAHİN, Funda ÇORAPÇIOĞLU, Zuhal KESKİN YİLDİRİM, Faruk Güçlü PINARLI, Nur OLGUN, Neriman SARI, Ayhan DAĞDEMİR, Derya ÖZYÖRÜK, Tuba EREN, Fatma Betül ÇAKIR, Başak ADAKLI AKSOY, Ceyhun BOZKURT, Elif GÜLER, Ali Aykan ÖZGÜVEN, Fatih ERBEY, Melda BERBER, Handan DİNÇASLAN, Emel ÜNAL, Mehmet KANTAR

**Affiliations:** 1 Department of Pediatric Oncology, Faculty of Medicine, Ankara Yıldırım Beyazıt University, Ankara Turkey; 2 Department of Pediatric Oncology, Faculty of Medicine, Ankara University, Ankara Turkey; 3 Department of Pediatric Oncology, Faculty of Medicine, Hacettepe University, Ankara Turkey; 4 Institute of Oncology, İstanbul University, İstanbul Turkey; 5 Department of Pediatric Hematology Oncology, Faculty of Medicine, İstanbul University, İstanbul Turkey; 6 Department of Pediatric Oncology, Dr. Sami Ulus Children’s Hospital, Ankara Turkey; 7 Department of Pediatric Oncology, Faculty of Medicine, Kocaeli University, Kocaeli Turkey; 8 Department of Pediatric Oncology, Faculty of Medicine, Atatürk University, Erzurum Turkey; 9 Department of Pediatric Oncology, Faculty of Medicine, Gazi University, Ankara Turkey; 10 Department of Pediatric Oncology, Faculty of Medicine, Dokuz Eylül University, İzmir Turkey; 11 Department of Pediatric Oncology, Dr. Abdurrahman Yurtaslan Oncology Training and Research Hospital, Ankara Turkey; 12 Department of Pediatric Oncology, Faculty of Medicine, Ondokuz Mayıs University, Samsun Turkey; 13 Department of Pediatric Oncology, Ankara Children’s Hematology and Oncology Training and Research Hospital, Ankara Turkey; 14 Department of Pediatric Oncology, Faculty of Medicine, Trakya University, Edirne Turkey; 15 Department of Pediatric Hematology Oncology, Faculty of Medicine, Bezmiâlem Vakıf University, İstanbul Turkey; 16 Department of Pediatric Hematology Oncology, Faculty of Medicine, Medeniyet University, İstanbul Turkey; 17 Department of Pediatric Hematology Oncology, Faculty of Medicine, Istinye University, Bahçelievler Medikal Park Hospital, İstanbul Turkey; 18 Department of Pediatric Oncology, Faculty of Medicine, Akdeniz University, Antalya Turkey; 19 Department of Pediatric Oncology, Faculty of Medicine, Celal Bayar University, Manisa Turkey; 20 Department of Pediatric Hematology Oncology, Faculty of Medicine, Koç University, Ankara Turkey; 21 Department of Pediatric Oncology, Faculty of Medicine, Ege University, İzmir Turkey

**Keywords:** Long term follow-up, survivorship, pediatric oncology, Turkey

## Abstract

**Aim:**

The main purpose of this study is to determine the current status of long-term follow-up (LTFU) for childhood cancer survivors and the challenges of LTFU for pediatric cancer survivors at pediatric oncology institutions in Turkey.

**Material and methods:**

A questionnaire was e-mailed to the directors of 33 pediatric oncology centers (POCs) registered in the Turkish Pediatric Oncology Group (TPOG). Of these 33 active TPOG institutions, 21 participated in the study and returned their completed questionnaires.

**Results:**

Only 1 of the 21 participating centers had a separate LTFU clinic. The remaining centers provided LTFU care for childhood cancer survivors at the pediatric oncology outpatient clinic. Of these centers, 17 (80.9%) reported difficulty in transition from the pediatric clinic to the adult clinic, 14 (66.6%) reported insufficient care providers, and 12 (57.1%) reported insufficient time and transportation problems. As neglected late effects, 16 (76.1%) centers reported psychosocial and getty job problems and 11 (52.3%) reported sexual and cognitive problems. None of the centers had their own LTFU guidelines for their daily LTFU practice.

**Conclusion:**

This study was the first to gain an overview of the needs of POCs and the gaps in survivorship services in Turkey. The results from this study will help to develop a national health care system and national guidelines for pediatric cancer survivors.

## 1. Introduction

As a result of advances in modern multidisciplinary treatment approaches and supportive care, the population of childhood cancer survivors has increased, with a survival rate of 80%. Although treatment regimens have been optimized to reduce immediate and late effects, long-term survivors of childhood cancers are at risk for treatment-related late effects throughout their lifespan. Approximately 73% of long-term survivors will have at least 1 chronic health problem within 30 years of their diagnosis, in which case, 42% are expected to have life-threatening conditions. The risk of developing these severe conditions is 8 times greater when compared to their siblings with no cancer [1–3].

Since the risk of many serious health problems can be reduced by prevention or early detection, survivors require systematic plans for periodic risk-adapted surveillance and prevention. In many developed countries, in response to a growing understanding of the potential treatment-related late effects, national long-term follow-up (LTFU) programs and guidelines have been developed for pediatric cancer survivors over the past 3 decades [1,4,5].

Over the past 20 years, Turkey has successfully introduced changes in key health system functions of organizations and governance, financing, resource management, and service delivery [6–8]. In parallel to the recent developments, the number of pediatric oncologists who were educated in developed centers has increased, and the procedures seen in multidisciplinary therapeutic approaches to childhood cancers has increased as well. Hence, the survival rates have increased dramatically [9,10]. As a result of the increase in cancer survival rates, it is crucial to identify current survivorship practices at pediatric oncology centers (POCs) across Turkey. There are no collective multicenter data related LTFU for childhood cancer survivors in Turkey. Some hospital-based late effects or secondary cancer publication data are available to date from Turkey [11–14]. The main purpose of this study was to determine the current status of LTFU for childhood cancer survivors and the challenges of LTFU for pediatric cancer survivors at pediatric oncology institutions in Turkey.

## 2. Methods

The study was conducted between October 2016 and February 2017. The descriptive survey instrument was developed by the investigators. The survey content and format were based on prior international surveys and guidelines [5,15–18]. A questionnaire was e-mailed to the directors of 33 POCs registered in the Turkish Pediatric Oncology Group (TPOG). Directors who did not respond to the first e-mail were sent a subsequent e-mail 4 weeks later. The surveys were collected over a period of 2 months. The questionnaire was organized with the following sections:

1. Description of the centers: historical time, yearly new patient number, follow-up period, and electronic medical record system.

2. Methods of follow-up for late effects: current clinical practice, determining the methods of LTFU, problems that cannot be evaluated in follow-up, and challenges for LTFU.

3. Transition problems of survivors to adult programs.

4. Educational problems of the hematology/oncology fellows (HOFs) about LTFU.

5. Suggestions for improvement of LTFU.

### 2.1. Statistics

Descriptive analyses were performed for all of the quantitative data and listed with frequencies and percentages for the categorical variables and medians and ranges for the quantitative variables. All statistical analyses were calculated using SPSS Statistics for Windows 17.0 (SPSS Inc., Chicago, IL, USA).

## 3. Results

### 3.1. Participating POCs

Of the 33 active TPOG institutions, 21 participated in the study and returned their completed questionnaires, with a final survey response rate of 63%. All of the POCs serv the health care needs of children, from infancy to adolescence. The POCs varied in terms of their size/number of beds and experience/historical time period. Table 1 displays the characteristics of the 21 POCs analyzed in this study. The median experience time as a POC and the median number of yearly new cases were 23 years (2–50 years) and 68 (8–300), respectively. Of the 21 centers that participated, 11 (52.3%) had an electronic record system, and 8 of these 11 centers had used a digital recording system since the year 2000. All of the POCs followed up the survivors annually. All of the POCs reported that the minimum duration of time that they cared for survivors was up to 18 years of age. On the other hand 10 (47.6%) and 4 POCs (19%) reported that this time was up to 25 years of age and to the end of life, respectively. Only 1 center reported that they had a separate LTFU clinic for survivors.

**Table 1:** Characteristics of 21 Turkish POCs.

Median historical time (years)	23.3 (range: 2–50)
Median number of the newly diagnosed patients/ year	67.9 (range: 8–300)
Median number of beds	15 (range: 3–32)
Number of POCs that follow survivors until their 18th birthday	21
Number of POCs that follow survivors until their 25th birthday	10
Number of POCs that follow survivors until the end of their life	4
Number of the POCs that followed survivors yearly	21
Number of POCs with a specialized LTFU clinic	1
Number of POCs with electronic records	11
Median lost follow up percentages	12% (range: 1–50)

### 3.2. LTFU programs

All of the centers reported that they followed up childhood cancer survivors. However, none of the centers used standardized, risk-based, survivor-focused guidelines. Of the centers, 8 reported that they usually recommend screening programs irrespective of the risk for late effects, and 13 used risk-based, but not standardized screening for late effects. Only 1 of the 21 participating centers had a separate LTFU clinic. This clinic was open 2 days a week. The remaining centers provided LTFU care for childhood cancer survivors at their pediatric oncology outpatient clinics.

All of the POCs reported at least 1 neglected late effect during LTFU. All of the POCs followed-up on cardiotoxicity, secondary cancer, and kidney toxicity with no problems. As for the neglected late effects, 16 (76.1%) POCs reported psychosocial and administrative problems, 11 (52.3%) reported sexual and cognitive late effects, 8 (38.1%) reported gonadal dysfunction, and 1 (4.7%) reported endocrinologic side effects. Details of the neglected late effects during LTFU are presented in Table 2.

**Table 2:** Neglected late effects of survivors in Turkish POCs during LTFU*.

Late effects	n	%
Psychological problems	16	76.1
Social problem (job, etc.)	16	76.1
Cognitive problems	11	52.3
Sexual dysfunction	11	52.3
School performance	11	52.3
Cosmetic problem	10	47.6
Oral health	9	42.8
Gonadal dysfunction	8	38.1
Genito-urinary problems	5	23.8
Neurological side effects	3	14.3
Ototoxicity	2	9.5
Endocrinologic side effects	1	4.7

*Some centers had more than one problem.

### 3.3. Barriers of LTFU

All of the POCs reported at least 1 challenge. Specifically, 17 (80.9%) POCs reported difficulty transitioning from the pediatric to adult clinic, 14 (66.6%) reported a lack of care providers, 12 (57.1%) reported a lack of time and transportation problems, and 8 (38%) reported a lack of providing knowledge to patients. Table 3 shows the barriers for LTFU.

**Table 3:** Barriers for Turkish POCs for the LTFU of pediatric cancer survivors.

Barriers	n	%
Lack of care providers	14	66.6
Lack of time	12	57.1
Financial problems of center	7	33.3
Lack of health insurance (after 18 years of age)	9	42.8
Transition problems of survivors from pediatric to adult clinics	12	57.1
Lack of providing knowledge to patients	8	38.1

All of the centers used the treatment summary report of the patients for the transition from the pediatric to adult center. This procedure was the routine transferring procedure of patients in all of the centers. Only 5 centers informed the adult departments about transferring by verbal communication.

### 3.4. Barriers for teaching pediatric HOFs about LTFU

Of the 21 centers, 17 had pediatric hematology/oncology fellowship training programs. Of those 17 POCs, 14 (66%) reported that they had insufficient education for LTFU, and 12 (85.7%) of those 14 centers asserted that the reason for the insufficient education was a lack of specialized LTFU clinics. Among the 9 centers out of a total 21 centers, 8 (57.1%) reported that the reason for the insufficient education was insufficient care providers and time, while it was a lack of education programs according to the 1 (7.1%) center.

## 4. Discussion

Cancer is a chronic disease that can cause lasting impact after treatment ends. As is the case for other middle-income countries, chronic diseases pose a major future challenge for Turkey. Therefore, there is a need for health policies that will confront this issue. Although the Ministry of Health has started various programs for the prevention and the control of chronic diseases, there is no routine surveillance and there is insufficient data on pediatric survivorship [19].

The TPOG and Turkish Pediatric Hematology Association established a web-based cancer registry in Turkey in 2002. Over the 14 years from 2002 to 2016, a total of 21,478 pediatric cancer cases were recorded. Survival rates for children have increased from 65% to 70% based on the latest information [9,10]. Unfortunately, there are no data about whether this large population has accessed LTFU services and the quality of the services available. The current study is the first to report national data for LTFU services in Turkey. The overall objective of this study was to gain a better understanding of the current state of LTFU in Turkey. The questionnaire survey was able to reach two-thirds of the POCs, located in 8 cities, that followed up most of the pediatric oncology patients in Turkey. Thus, the findings reflected the latest results for Turkey. The results of this study will help to improve the survivorship services in Turkey via preparing a short guideline to standardized care with an easy risk-adapted formula.

The most important finding of this study was that pediatric cancer survivorship has not flourished in Turkey because only 1 center reported having a separate LTFU clinic. In the remaining 20 centers, LTFU was provided by the pediatric oncologists in the outpatient clinics. There is an urgent need for specialized LTFU clinics, where medical and allied health professionals, such as social workers, clinical psychologists, and rehabilitation specialists, work in Turkey. Only 4 centers reported providing caring for survivors until the end of their lives. However, the other centers provided care for childhood cancer survivors and patients younger than 30 years of age. In Turkey, the relevant law, which was revised in 2018, states that diseases that start in childhood and require diagnosis, treatment, and follow-up through adulthood can be cared for and followed up by pediatric subspecialists until the age of 23 [20].

This loss of survivors to the lack of follow-up means that such patients will receive ongoing medical care from a clinician other than a pediatric oncologist, and they will not be monitored for late effects. Moreover, these responses suggest inconsistency with legislation. In addition, about half of the centers had no electronic medical records to monitor the patients.

POCs have several challenges, and herein, 80.9% of the POCs reported difficulties transitioning from the pediatric to adult clinics, 66.6% reported a lack of health care providers, and 57.1% reported a lack of time. The results of the study reflected that one of the most important barriers was insufficient healthcare system support that established a multidisciplinary coordination of care between the pediatric and adult medical subspecialists. Another factor that promoted these challenges was the absence of a formal adult follow-up program in Turkey. These barriers have been reported repeatedly in the literature, to implement survivorship care, even in developed countries with an organized healthcare system [15,16,21–24].

In most developing countries, such as Turkey, common practice is to focus on the monitoring of cancer recurrence and symptoms directly related to the treatment. The results herein showed that, generally, POCs do not provide sufficient LTFU. All of the centers agreed on the necessity of risk-based national guidelines for LTFU.

In addition, this study revealed that there were many barriers in the process of teaching pediatric HOFs for LTFU. The main barrier was the lack of specialized LTFU clinics. Nathan et al. reported a lack of time for training to learn about the late effects as the most significant barrier to provide survivorship [17].

In many developed countries, in response to a growing understanding of the potential treatment-related late effects, national LTFU programs have been created for pediatric cancer survivors over the past 3 decades. These programs are influenced by political, economic, sociocultural, and institutional factors. For developing countries like Turkey, the process of providing care is often weak and continuously changing. In Turkey, reforms have focused on access to primary health care services [6–8]. Unfortunately, there has been no improvement in this area. In the current Turkish health care system, the question of responsibility for the care of cancer survivors among health care providers remains unclear.

How can these barriers be overcome? As members of the TPOG, we know that we have to develop short and long term solutions for these problems. After developing a handy survivorship guideline according to the needs of the centers, it will be important to share a national program that determines specific information about what services are provided, how they are provided, and by whom and for whom they are provided. In 2005, family medicine-centered primary health-care services were developed in all provinces of Turkey [6]. In order to effectively manage pediatric cancer survivors, it is necessary to ensure communication between pediatric oncologists and family physicians through treatment summaries and survivor care plans. Financial, staff, and infrastructure support is needed to establish such a program, thus cooperation with politicians is needed. The role of the government is to establish principles, and set the political agenda for survivorship services. Even in developed countries, in which LTFU systems that have been established long ago, many difficulties in clinical practice have been reported. Therefore, a simple and practical system should be developed to address these barriers. The publication of new guidelines after a workshop study with these centers has been planned.

This study had several limitations. The main limitation of this study was the self-reporting of the data by the centers themselves. Another limitation was that the study was conducted among POCs; hence, some patients with leukemia might not have been included in this group because they were followed up by pediatric hematology centers. Furthermore, it was not possible to compare the data with the one from other countries with similar problems due to the limited literature. Despite these limitations, this study was the first to gain an overview of the needs of POCs and the gaps in survivorship services in Turkey. The results from this study will help to develop a national health care system and national guidelines for pediatric cancer survivors.

In conclusion, reports from 21 POCs in Turkey, in 2017, were reviewed and analyzed for survivorship practices. Many of the barriers of survivorship care included issues of access (access to what?), quality, and funding. These barriers were similar to those identified for LTFU in other developing countries and will establish the basis of the planned LTFU guideline study.
